# The complete chloroplast genome of *Gleditsia sinensis* and *Gleditsia japonica*: genome organization, comparative analysis, and development of taxon specific DNA mini-barcodes

**DOI:** 10.1038/s41598-020-73392-7

**Published:** 2020-10-01

**Authors:** Wei Tan, Han Gao, Weiling Jiang, Huanyu Zhang, Xiaolei Yu, Erwei Liu, Xiaoxuan Tian

**Affiliations:** State Key Laboratory of Component-based Chinese Medicine, Tianjin University of Traditional Chinese Medicine, Poyang Lake Road 10, Tianjin, 301617 China

**Keywords:** Genomics, Comparative genomics, Medical genomics

## Abstract

Chloroplast genomes have been widely considered an informative and valuable resource for molecular marker development and phylogenetic reconstruction in plant species. This study evaluated the complete chloroplast genomes of the traditional Chinese medicine *Gleditsia sinensis* and *G. japonica,* an adulterant of the former. The complete chloroplast genomes of *G. sinensis* and *G. japonica* were found to be of sizes 163,175 bp and 162,391 bp, respectively. A total of 111 genes were identified in each chloroplast genome, including 77 coding sequences, 30 tRNA, and 4 rRNA genes. Comparative analysis demonstrated that the chloroplast genomes of these two species were highly conserved in genome size, GC contents, and gene organization. Additionally, nucleotide diversity analysis of the two chloroplast genomes revealed that the two short regions of *ycf1*b were highly diverse, and could be treated as mini-barcode candidate regions. The mini-barcode of primers ZJ818F-1038R was proven to precisely discriminate between these two species and reflect their biomass ratio accurately. Overall, the findings of our study will shed light on the genetic evolution and guide species identification of *G. sinensis* and *G. japonica*.

## Introduction

*Gleditsia* (honey locust) is a genus comprising 13 species of the Caesalpinioideae subfamily and Fabaceae family^1^. The honey locust is native to North America and Asia, and a majority of the species diversity is found in eastern Asia^2^. Previous investigation on plants of the *Gleditsia* genus showed a variety of bioactivities, including anti-tumor, anti-inflammatory, anti-hyperlipidemic, anti-allergic, and analgesic^3^. Therefore, plants of the *Gleditsia* species have been widely used for centuries in local and traditional medicine^1^. For example, *G. japonica* has long been known to be a diuretic and an expectorant^4^, and the medicinal value of *G. sinensis* is documented in various editions of the Pharmacopoeia of the People’s Republic of China, from 1965 to 2015^5,6^. Thorns of the honey locust, known as ‘Zao Jiao Ci’, are used in traditional oriental medicine as an efficacious therapeutic agent for the treatment of carbuncle, cancers, skin diseases, and suppuration^7,8^. Some components of *G. sinensis* also constitute some patent medicines, like the *Gleditsia* pill and Wang Bi capsules.

Angiosperm chloroplast genomes are key organelles for photosynthesis and carbon fixation^9^. The chloroplast genomes are valuable resources for molecular identification and phylogenetic studies because of a series of superiorities including the compact size, less recombination, maternal inheritance, self-replication, high copy number, and moderate substitution rates^10–13^. Comparative analysis of the chloroplast genomes of closely related species is crucial for grasping various aspects of genome evolution, focused on the structural variations and gene losses^14^. However, only one chloroplast genome of the genus *Gleditsia* has been reported so far^15^*,* which highly limits our understanding of the evolution and phylogeny of *Gleditsia*.

Due to their similar morphologic characteristics, unintentional adulteration of *G. sinensis* and *G. japonica* frequently occurs in China^16^. The current methods for distinguishing between the two include chemical^17^, morphological, and microscopic techniques^16^. However, precise discrimination of processed material of the species is often challenging^18^. DNA barcoding is a molecular marker technology that can accurately and rapidly identify different species and does not require any specialized training or evaluation of obvious morphological characteristics^19,20^. Previous studies have used the *ndhF* and *rpl16* gene sequences^21^ of 11 species of the *Gleditsia* genus for phylogenetic and biogeographic analysis in *Gleditsia*. Besides, the *trnL-trnF* intergenic spacer and the *trnL* intron^22^ have also been used to distinguish between five *Gleditsia* species. Further, some researchers sequenced *psbA-trnH*^23^ and *matK*^24^ to identify *G. sinensis.* However, the fruits and thorns of *G. sinensis,* which are used in traditional medicine and several Chinese patent medicines usually undergo varying degrees of DNA degradation during harvesting, storage, and processing. Notably, the amplification of the full-length barcode in the degraded samples is challenging^25^. At the same time, the common markers (438–2098 bp) could introduce serious bias in biomass estimation when applied for metabarcoding analysis of degraded DNA mixtures^26^. To mitigate the problem of DNA degradation and quantitative inaccuracy, numerous studies have indicated that mini-barcodes (generally ≤ 200 bp) can be used instead of the traditional full-length barcodes, as they distinguish between limited species^27–29^. Therefore, our aim is to develop a mini-barcode that can be used for the quantitative identification of *G. sinensis* and its counterfeit *G. japonica.* For seed plants, such barcodes are identified by screening the chloroplast genome, owing to its advantages stated above^20,30^.

In this study, we sequenced the complete chloroplast genome of *G. sinensis* and *G. japonica*, which have been less studied in previous researches. The specific aims of the present study were to: (1) obtain the complete chloroplast genomes of *G. sinensis* and *G. japonica*; (2) carry out a comparative analysis of the chloroplast genomes of these two species; (3) evaluate the monophyletic and systematic position of *Gleditsia* by reconstructing phylogenetic relationships of the 152 species of the Fabaceae family; (4) detect the suitable mini-barcode region for species identification of these two species; (5) validate the quantitative capacity of mini-barcode primers by meta-barcoding. Our results will provide valuable data for accurate species-level discrimination between *G. sinensis* and *G. japonica* and help preserving the quality of *G. sinensis* as an important Chinese medicine.

## Results

### Complete chloroplast genome features and organization of *G. sinensis* and *G. japonica*

**Figure 1:**
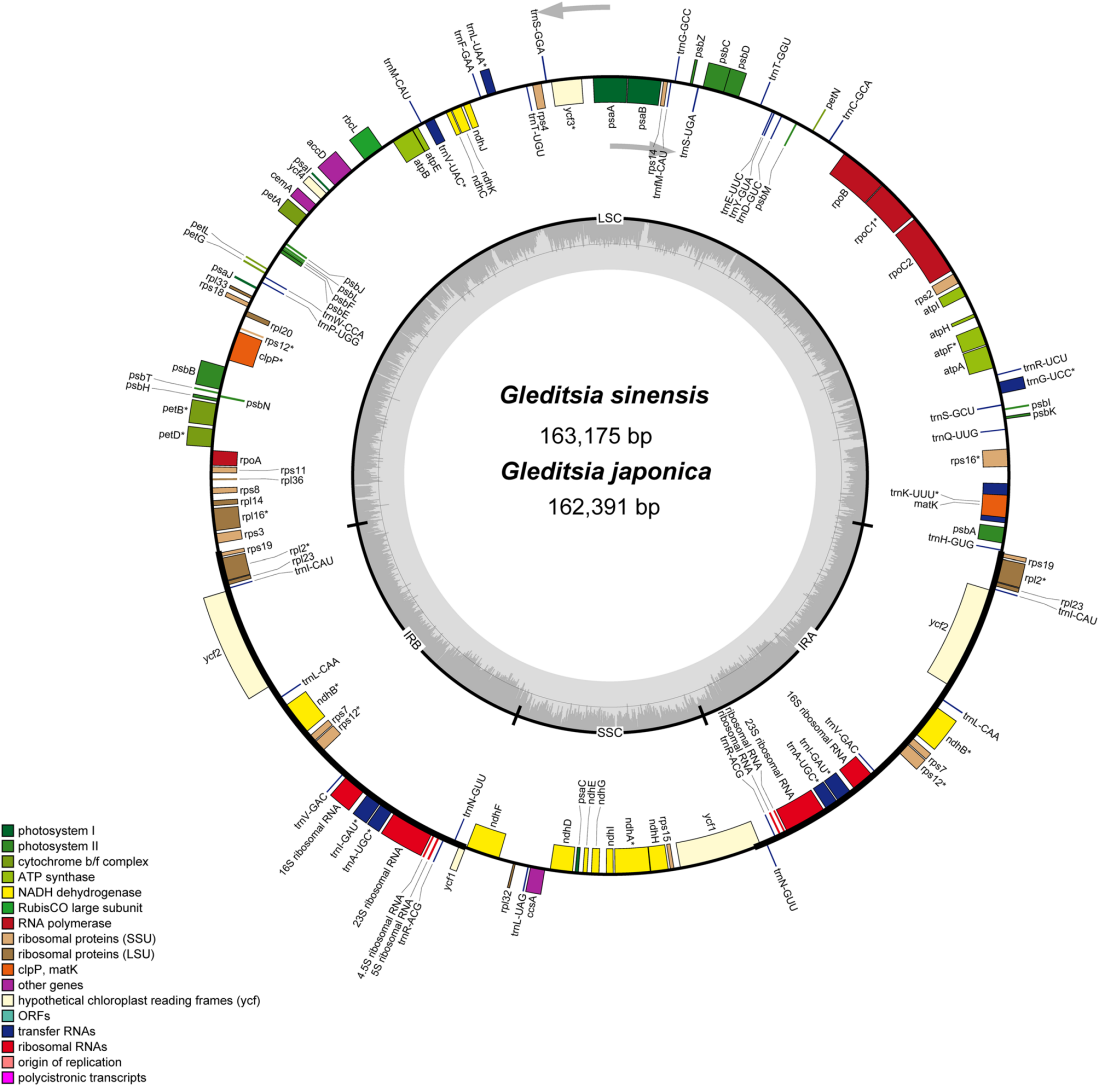
Gene map of the complete chloroplast genomes of the two *Gleditsia* species. Genes on the inside of the circle are transcribed clockwise, whereas those outside are transcribed counter-clockwise. The dark gray and light gray shading within the inner circle correspond to the percentages of G + C and A + T contents, respectively.

As shown in Fig. 1, the two *Gleditsia* species displayed similar quadripartite structures, including a pair of inverted repeats in the IR regions (IR), one large single-copy (LSC) region, and one small single-copy (SSC) region. The chloroplast genome sizes of *G. sinensis* and *G. japonica* were 163,175 bp and 162,391 bp, respectively. Each chloroplast genome encoded 111 unique genes, including 77 coding sequences, 30 tRNA and 4 rRNA genes. The G + C content of the *G. sinensis* chloroplast genome was 35.6%, which demonstrated congruence with that of *G. japonica* (35.5%) (Table 1). Furthermore, *infA* and *rpl22* genes were lost in each species because of transfer to the nucleus ^31,32^ (Table 2). The *rps12* gene was spliced into two transcripts, with exon 1 in the LSC region and exons 2 and 3 in the IR region, which is consistent with that in the previous studies^33,34^. 15 genes (*rpl16, rpl2, rps16, rpoC1, trnA-UGC, trnG-UCC, trnI-GAU, trnK-UUU, trnV-UAC trnL-UAA, ndhA, ndhB, petB, petD,* and *atpF*) contained one intron, and two genes, i.e., *clpP, ycf3* harbored two introns (Table 2).

**Table 1:** Comparison of the chloroplast genome organization of the two *Gleditsia* species.

Genome features	*Gleditsia sinensis*	*Gleditsia japonica*
Total reads (bp)	29,589,646	28,279,048
Size (bp)	163,175	162,391
LSC (bp)	91,540	91,449
SSC (bp)	19,249	19,449
IR (bp)	26,193	25,866
Number of genes	111	111
Protein-coding genes	77	77
tRNA genes	30	30
rRNA genes	4	4
Total G + C content (%)	35.6	35.5

**Table 2:** Gene contents in the chloroplast genomes of the two *Gleditsia* species.

Gene category	Gene groups	Names of genes
Self-replicating	Large subunit of ribosome (LSU)	*rpl14, rpl16*^*a*^*, rpl2*^*a*^* (2), rpl20, rpl23(2), rpl32, rpl33, rpl36*
	Small subunit of ribosome (SSU)	*rps11, rps12*^*c*^* (2), rps14, rps15, rps16*^*a*^*, rps18, rps19 (2), rps2, rps3, rps4, rps7 (2), rps8*
	DNA dependent RNA polymerase	*rpoA, rpoB, rpoC1*^*a*^*, rpoC2*
	rRNA genes	*rrn16* (2)*, rrn23 *(2)*, rrn4.5 *(2)*, rrn5 *(2)
	tRNA genes	*trnA-UGC*^*a*^*(2), trnC-GCA, trnD-GUC, trnE-UUC,* *trnF- GAA, trnfM-CAU, trnG-GCC, trnG-UCC*^*a*^*,* *trnH-GUG, trnI-CAU (2), trnI- GAU*^*a*^*(2), trnP-UGG* *trnK-UUU*^*a*^*, trnL-CAA (2), trnL-UAA*^*a*^*, trnL-UAG, trnM- CAU, trnN-GUU (2), trnS-GCU, trnS-GGA,* *trnS-UGA, trnT-GGU, trnT-UGU, trnV-GAC (2),* *trnV-UAC*^*a*^*, trnW-CCA, trnY-GUA, trnQ-UUG,* *trnR-ACG (2), trnR-UCU*
Photosynthesis	Photosystem I	*psaA, psaB, psaC, psaI, psaJ*
	Photosystem II	*psbA, psbB, psbC, psbD, psbE, psbF, psbH, psbI,* *psbJ, psbK, psbL, psbM, psbN, psbT, psbZ*
	NADH dehydrogenase	*ndhA*^*a*^*, ndhB*^*a*^* (2), ndhC, ndhD, ndhE, ndhF,* *ndhG, ndhH, ndhI, ndhJ, ndhK*
	Cytochrome b/f complex	*petA, petB*^*a*^*, petD*^*a*^*, petG, petL, petN*
	Subunits of ATP synthase	*atpA, atpB, atpE, atpF*^*a*^*, atpH, atpI*
	Large subunit of Rubisco	*rbcL*
Other genes	Protease	*clpP*^*b*^
	Maturase	*matK*
	Envelop membrane protein	*cemA*
	Subunit of acetyl-CoA	*accD*
	C-type cytochrome synthesis gene	*ccsA*
Unknown function	Proteins of unknown function	*ycf1 (2), ycf2 (2), ycf3*^*b*^*, ycf4*

^a^genes containing a single intron.

^b^genes containing two introns.

^c^ genes divided into two independent transcription units.

### Repeated sequence analysis

**Figure 2:**
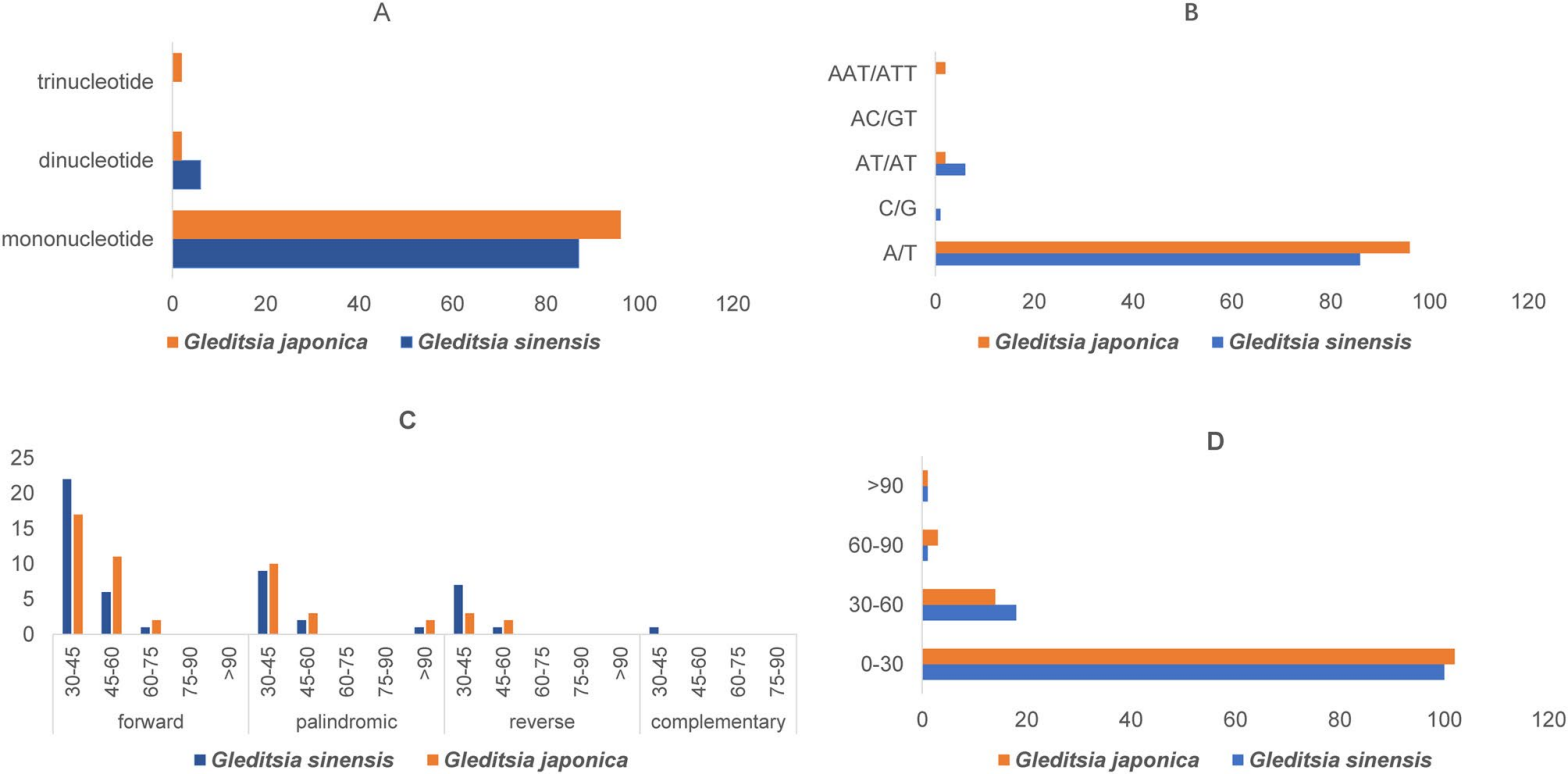
Analysis of repeated sequences in the two *Gleditsia* species. (**A**) The numbers of different SSR types, including mononucleotide, dinucleotide, and trinucleotide; (**B**) Number of different SSR repeat units. (**C**) Frequency of repeat sequences in the two chloroplast genomes as determined by REPuter; (**D**) Frequency of tandem repeat sequences by length.

Simple sequence repeats (SSR) that are highly polymorphic at the intra-specific level could be treated as molecular markers in population genetics and evolutionary studies^30,35^. Besides, mononucleotide SSR markers derived from chloroplast genomes form an excellent basis for studying the female lineage of polyploid species, because of their uniparental inheritance and non-recombination during sexual reproduction^36,37^. In this study, a total of 93 microsatellites were identified in the chloroplast genome of *G. sinensis*, including 87 mononucleotide and 6 dinucleotide SSR. Meanwhile, a total of 100 SSR were detected in the whole chloroplast genome of *G. japonica,* comprising 96 mononucleotide, 2 trinucleotide, and 2 tetranucleotide SSR (Fig. 2A). The most abundant microsatellites were mononucleotide repeats (183), accounting for about 96.45% of the total SSR (193). Among all mononucleotides, about 99.45% were A/T (182), whereas C/G (1) only accounted for 0.55% (Fig. 2B). This result is congruent with the previous observation that chloroplast genome SSR are generally composed of A/T, and rarely C/G^38^. The second abundant SSR were dinucleotide repeats (8), followed by trinucleotide repeats (2), while tetranucleotide, hexanucleotide and pentanucleotide repeats were not found. Our findings suggest that mononucleotide repeats may contribute to more genetic variations than other SSR, which is consistent with previous study findings^35^.

According to a previous report, the contribution of longer repeat sequences to genome rearrangement and recombination is more significant than that of shorter SSR^39^. In this study, dispersed repeat segments in the two *Gleditsia* species were analyzed by REPuter. The results revealed four types of repeated sequences (forward, reverse, palindromic, and complementary) in *G. sinensis,* but no complementary repeats were detected in *G. japonica*. Figure 2C exhibits that most of these repeats were forward and palindromic, with a length range of 30–45 bps in the two *Gleditsia* species. Tandem repeats in both species were 120, and the majority of these repeats were between 0 and 30 bp in length (Fig. 2D). In general, the repeats identified in this study will provide valuable information for the study of population relationships in the *Gleditsia* species.

### Analysis of codon preference

**Figure 3:**
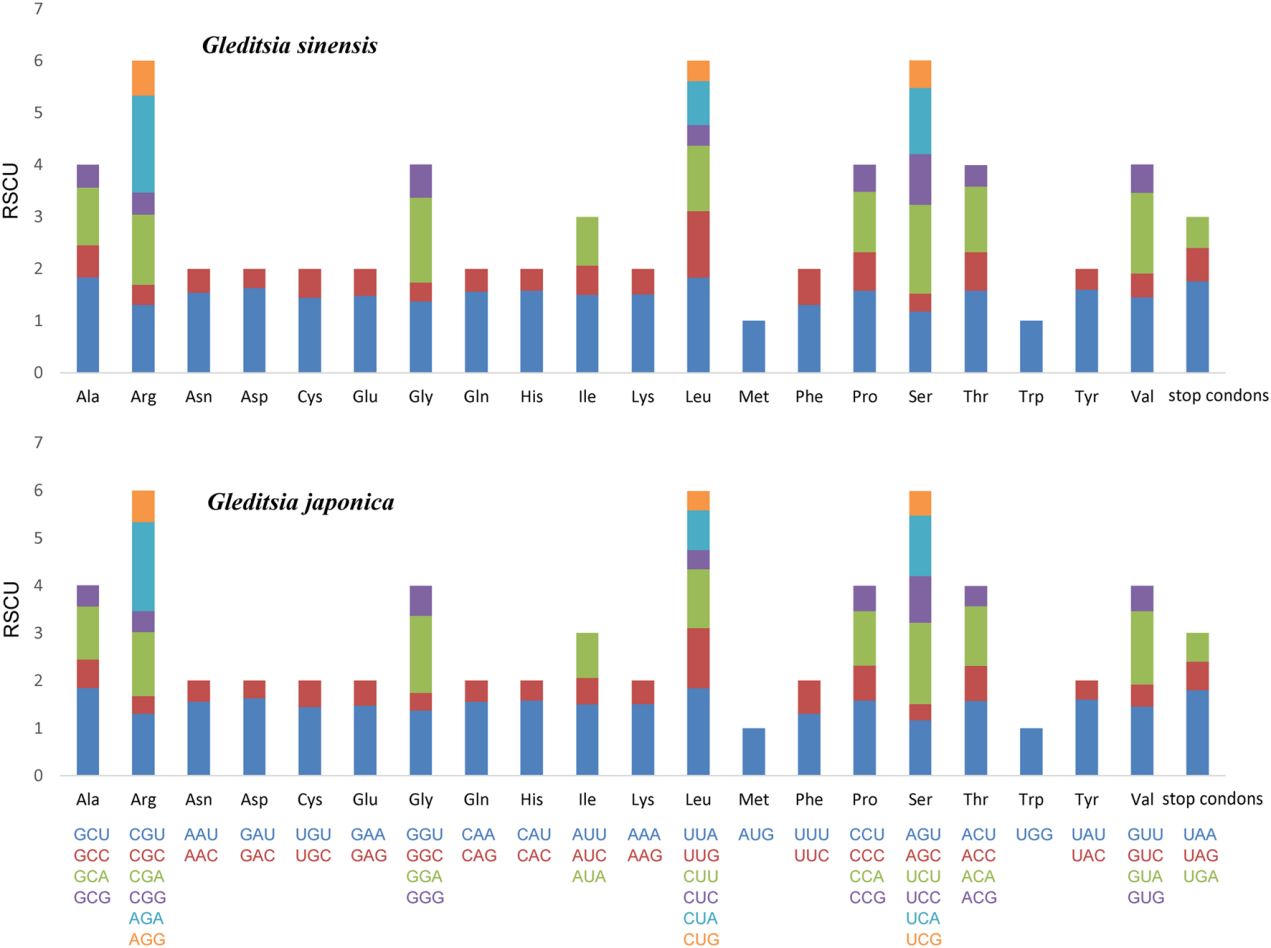
Codon contents of the 20 amino acids and stop codons in all protein-coding genes in the chloroplast genomes of the two *Gleditsia* species.

As codon usage plays a vital role in shaping chloroplast genome evolution^40^, the relative synonymous codon usage frequency (RSCU) between *G. sinensis* and *G. japonica* was calculated using the protein-coding sequences in the chloroplast genomes. The protein sequences contained 26,239 and 26,249 codons, respectively, including stop codons. As shown in Fig. 3 and Supplementary Tables S1–S2, leucine was encoded by the highest number (average = 10.56% and 10.45%) of codons, while cysteine (average = 1.193% and 1.192%) was the least encoded in *G. sinensis* and *G. japonica,* respectively. In addition, most of the amino acids showed codon bias except methionine (AUG) and tryptophan (UGG) (RSCU = 1), which indicated no codon preferences. Similar to the chloroplast genomes of other higher plant^40,41^, nearly all codons of the two species with high RSCU values (RSCU > 1.3) showed a high A/U preference in the third codon. This codon usage pattern may be driven by a composition bias for high proportions of A/T^41^. Meanwhile, we found that the chloroplast genome codon usage of these two species was very similar (Fig. 3). In general, the present results revealed the relative conservation of the chloroplast genomes of *G. sinensis* and *G. japonica.*

### RNA editing site prediction

RNA editing can participate in the post-transcriptional regulation of chloroplast genomes by nucleotide insertion, deletion, or substitution, which provides an effective way of creating transcriptional and translational diversity^42,43^. A total of 52 and 53 RNA editing sites were predicted in 18 chloroplast genes of *G. sinensis* and *G. japonica*, respectively (Supplementary Tables S3–S4). Among these sites, the highest frequency of amino acid conversion involved serine (S) to leucine (L), which concurs with previous investigations in the chloroplast genomes of higher plant^44^. As previously reported, the number of shared editing sites increases in closely related taxa^45^. In this study, we found that *G. sinensis* shared editing sites with *G. japonica*, indicating that RNA editing was evolutionary conserved.

### Comparison of the chloroplast genome structures of the two *Gleditsia* species

**Figure 4:**
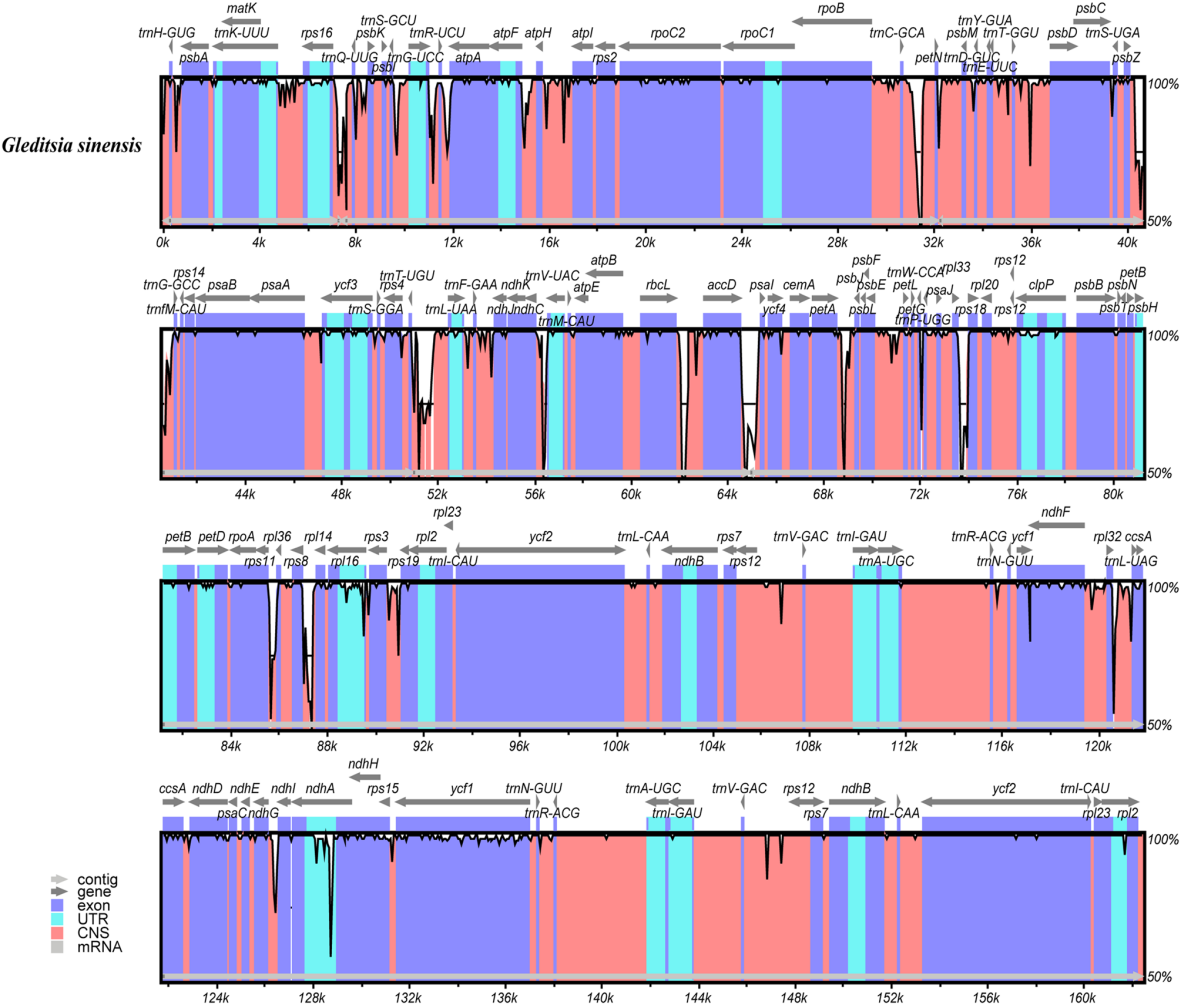
Visual alignment of the chloroplast genomes of the two *Gleditsia* species. VISTA-based identity plot showing sequence identity among the two species, using *G. japonica* as reference.

Multiple sequence alignment of the chloroplast genomes of the two *Gleditsia* species was performed by mVISTA, using the annotated chloroplast genome sequence of *G. japonica* as reference. The result (Fig. 4) showed that the genomes of the two species are highly conserved, with some degree of divergence. Comparative analysis by MAUVE showed that the chloroplast genome structures of the two *Gleditsia* species were identical (Supplementary Fig. S1).

### Phylogenetic analysis

**Figure 5:**
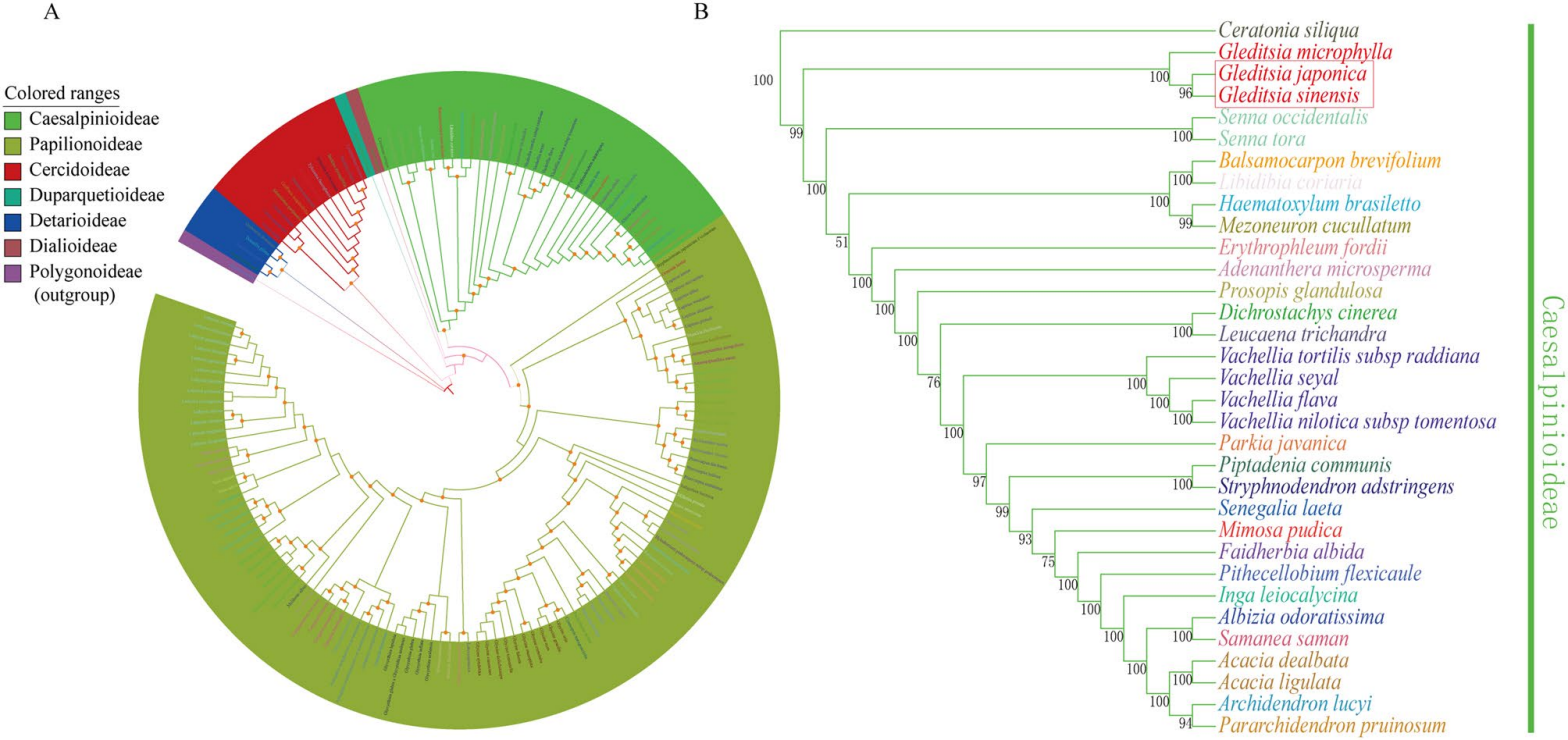
Phylogenetic tree reconstruction of the 155 species inferred from maximum likelihood (ML) based on 75 protein-coding genes of the complete chloroplast genomes. (**A**) Phylogenetic relationship of Leguminosae, the orange dots at nodes on the tree indicate bootstrap values (= 100). (**B**) Phylogenetic relationship of Caesalpiniaceae, numbers at nodes on the tree represent bootstrap values.

Recent advances in high-throughput sequencing provide large amounts of data, which could improve phylogenetic resolution^41,46^. Furthermore, chloroplast genomes have proven highly reliable in inferring the phylogenetic relationships between numerous plant groups^47^. In this study, phylogenetic relationships in the Leguminosae family were reconstructed based on 75 protein-coding genes from 155 legume species. The phylogenetic tree was divided into six subfamilies, which accorded well with the Fabaceae classification system revised in 2017^48^ (Fig. 5), all the nodes were moderately or highly supported. In our study, three species of the *Gleditsia* genus formed a monophyletic clade with strong bootstrap values. The phylogenetic position of *Gleditsia* is consistent with previous study reports^22,48–50^. Our data will be a useful resource for molecular phylogeny studies within Leguminosae, particularly regarding the role of *G. sinensis and G. japonica* in plant systematics and evolution.

### Analysis of sequence divergences and DNA mini-barcodes

**Figure 6:**
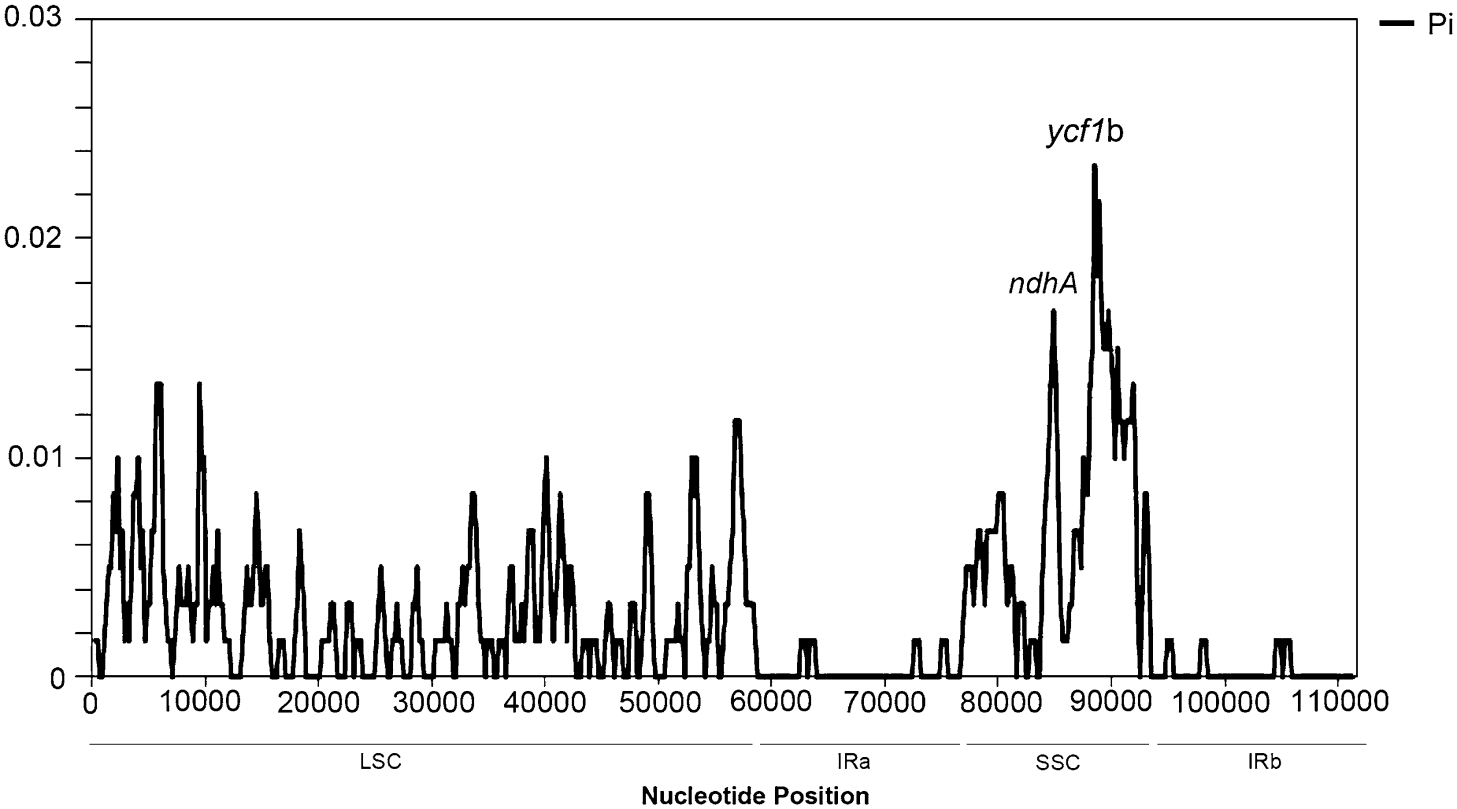
Nucleotide diversity (Pi) based on sliding window analysis of *G. sinensis* and *G. japonica* using 130 chloroplast genes. X-axis, the position of the midpoint of a window; Y-axis, nucleotide diversity of each window.

Highly variable DNA regions of chloroplast genomes could be used to distinguish between closely related species^51^. In this study, a total of 130 genes shared between the two *Gleditsia* species were used to estimate nucleotide diversity. The results showed that nucleotide variability (Pi) of the two species ranged from 0.00001 to 0.02333 (Fig. 6), with a mean of 0.00210. Meanwhile, the SSC region showed the highest levels of divergence. In this region, *ycf1*b exhibited remarkably higher Pi values (0.02333), and was, thus, treated as a potential marker for distinguishing between these two species. Two primer pairs were designed within *ycf1*b using Primer3^52^ (Table 3), and amplicons from the two *Gleditsia* species were compared with other plant universal marker regions of *rbcL*^53^, and *ndhF, rpl16*^21^*, trnL-trnF, trnL* intron^22^, *psbA-trnH*^23^ and *matK*^24^ as described in previous studies. As Table 4 indicates, two short regions of *ycf1*b (189 bp and 134 bp, respectively) had more variable sites. This result is consistent with the previous report that *ycf1* is one of the most promising chloroplast DNA barcodes for land plants^54^. In ginsengs (another Chinese medicinal herb), *ycf1*b also has 100% discriminating power for closely related species^20^.

**Table 3:** Two pairs of primers of *ycf1*b mini-barcodes.

Primer name	ZJ818F-1038R	ZJ1118F-1287R
Forward primer sequence 5′ to 3	CTTCCAAAACGAAGAT	CTAGTTTGTCAACTTTTC
Reverse primer sequence 5′ to 3	AGCATTTTCAAATCGA	AAATTCCTTATCTAGAGC
Amplicon size (bp)	221	170
Sequence size excluding primers (bp)	189	134

**Table 4:** Features of nine marker regions in these two *Gleditsia s*pecies.

Number	Marker region	Aligned length (bp)	Variable sites		K2P	References
			Number	%		
1	*ycf1b-*ZJ818F-1038R	189	6	3.175	0.03260	This paper
2	*ycf1b-*ZJ1118F-1287R	134	6	4.478	0.04690	This paper
3	*matK*	1500	8	0.533	0.00536	Wojciechowski et al
4	*rbcL*	703	4	0.569	0.00571	Chen et al
5	*trnH-psbA*	438	5	1.142	0.01240	Liu et al
6	*ndhF*	2098	11	0.524	0.00527	Schnabel et al
7	*rpl16* intron	1122	11	0.980	0.01040	Schnabel et al
8	*trnL* intron	566	5	0.883	0.00890	Herendeen et al
9	*trnL-trnF*	499	7	1.402	0.01440	Herendeen et al

### Validation of the quantitative capacity of mini-barcode primers by metabarcoding

**Figure 7:**
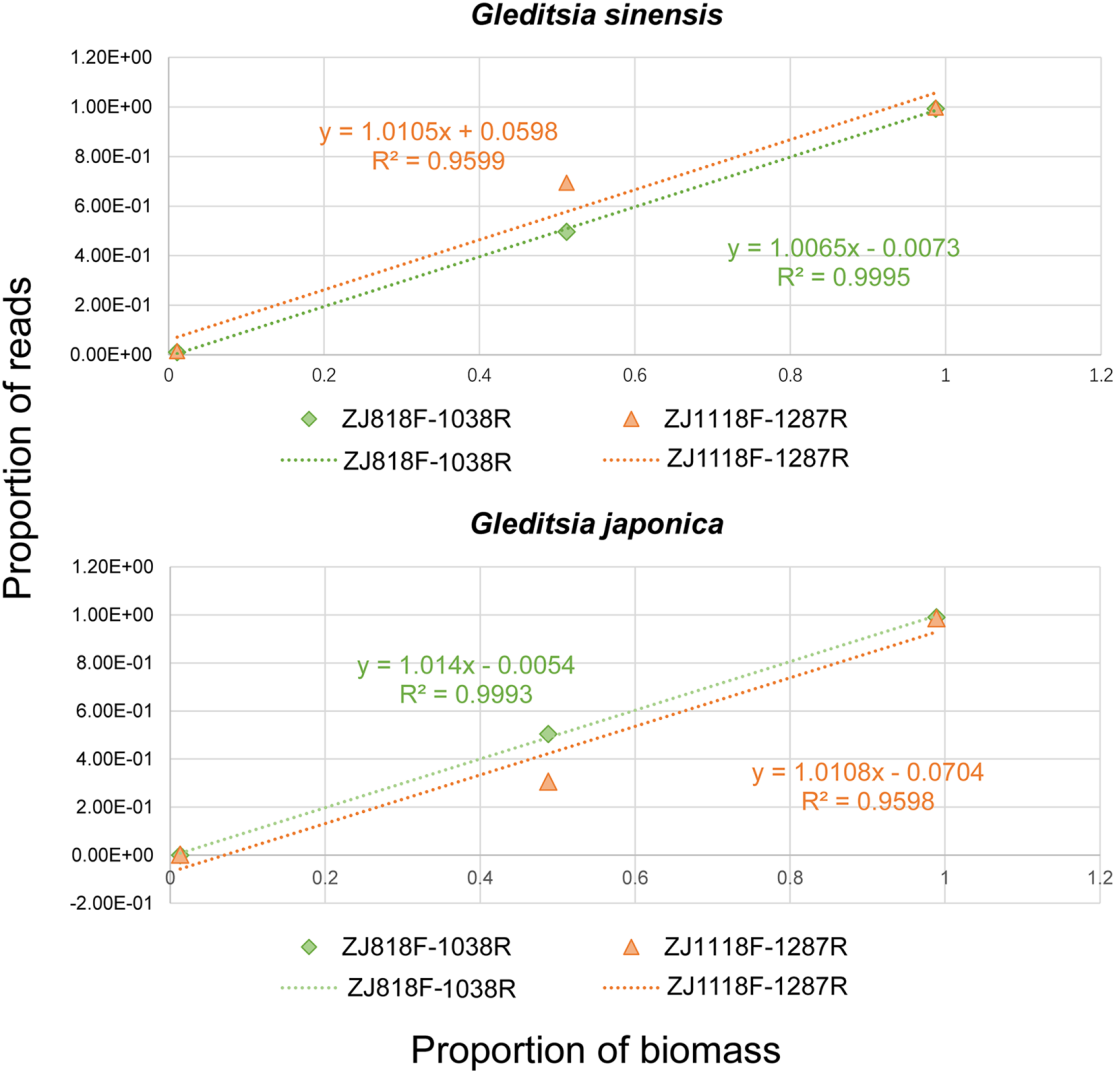
The relationship between biomass and read counts of the products amplified by the two primer pairs (ZJ818F-1038R and ZJ1118F-1287R) in the two species. X-axis, the proportion of biomass; Y-axis, the proportion of reads.

The DNA of three artificial mocks consisting of two *Gleditsia* species was extracted, PCR conducted using the two primer pairs described above, and the respective amplicons were submitted for high-throughput sequencing. The raw data consisted of 1,549,811 reads, of which 1,394,781 high-quality reads were retained after denoising and removal of low-quality and chimeric sequences with DADA2. Subsequently, we generated 3 (product of ZJ818F-1038R) and 5 (product of ZJ1118F-1287R) reliable amplicon sequence variants (ASV) for each amplicon, respectively. In ZJ818F-1038R, all ASVs could be identified as either *G. sinensis* or *G. japonica*. For ZJ1118F-1287R, 3 ASVs could be identified, accounting for 99.9% of the total sequences (Supplementary Tables S5–S6). As expected, both primer sets could recover species with very low abundance (1.1%). The results exhibited that the two species presented positive relationships between biomass and read counts, especially for the mini-barcode of primer ZJ818F-1038R, with significant correlations (Fig. 7). Overall, we expect that this mini-barcode can be used for the quantitative identification of the two *Gleditsia* species in actual production.

### Evaluation of the efficiency of the mini-barcode of primers ZJ818F-1038R in identifying processed medicinal materials

PCR analysis showed that primer ZJ818F-1038R had an excellent amplification efficiency of processed medicinal herbs and Chinese patent medicine (Supplementary Fig. S2). Sanger sequencing of the amplicons from Da Zao Jiao, Zao Jiao Ci, and Wang Bi capsules identified all the three samples as *G. sinensis*, with similarities of 99.47%, 100%, 100%, respectively (Supplementary Table S7).

## Conclusions

In this study, we assembled and characterized the complete chloroplast genomes of *G. sinensis* and *G. japonica.* The basic gene information, RNA editing sites, and codon usage patterns were revealed. A total of 93 and 100 SSR were identified in the complete chloroplast genomes of *G. sinensis* and *G. japonica*, respectively. Comparative analysis showed that the two *Gleditsia* species have similar chloroplast genome structures and showed an overall high degree of synteny*.* Also, we found that *ycf1*b was the most variable region among 130 genes, and could, thus, be treated as a potential DNA mini-barcode marker. Quantitative analysis based on *ycf*1b markers using the metabarcoding method was conducted, and the result showed that primer ZJ818F-1038R have more accurate quantitative ability*.* Overall, the findings of our study will shed light on the genetic evolution and species identification of *G. sinensis* and *G. japonica*.

## Discussion

With the increased application of high-throughput sequencing technology, the number of characterized chloroplast genomes of angiosperms is increasing rapidly^55^. In this study, we found that the two newly sequenced *Gleditsia* species have similar quadripartite structure and gene contents to the published chloroplast genomes of other members of the Caesalpinioideae sub-family^15,56–58^. According to phylogenetic analysis results, the genus *Gleditsia* forms a monophyletic clade with strong bootstrap values, which is consistent with the results of previous studies^22,48–50^. The Caesalpinioideae subfamily belongs to the Fabaceae family, which is divided into three long-recognized subfamilies, Caesalpinioideae, Mimosoideae, and Papilionoideae^59^. However, phylogenetic analysis based on *matK* genes^48^, nuclear genes (CYC2 genes)^50^ and chloroplast genomes^15,57^ suggests that the Fabaceae family should be divided into six subfamilies: Duparquetioideae, Cercidoideae, Detarioideae, Dialioideae, Caesalpinioideae, and Papilionoideae, which is now accepted widely. In our study, 155 Fabaceae species were used to construct phylogenetic trees based on chloroplast protein-coding genes. Our result is consistent with the recent phylogenomic analyses of Fabaceae.

Compared to traditional methods, DNA barcoding can be applied to accurately identify *G. sinensi*s and its adulterant, *G. japonica*. DNA barcodes refer to relatively short fragments of DNA with substantial genetic variation, which can be standardized, easily amplified, and representative^55^. DNA degradation frequently occurs during the production of natural medicine, which can decrease the efficiency of PCR^29^. According to the previous studies^20,25^, the usage of short DNA fragments, such as mini-barcodes, can effectively mitigate this problem. Additionally, with the advancement in high-throughput sequencing and metabarcoding, the development of mini-barcode primers is encouraged, which will, in turn, improve the efficiency of taxon discovery and identification^60,61^, especially in mixed samples. In the present study, metabarcoding was performed via sequencing of two mini-barcode amplicons, and quantitative assessments were conducted on three artificial communities. Taberlet et al.^62^ have suggested that the quantitative ability of metabarcoding remains to be tested, due to primer bias. However, the PCR product of primer ZJ818F-1038R used in this study showed highly significant correlations between read counts and biomass, thus good quantitative ability. Subsequently, the mini-barcode of primer ZJ818F-1038R was found useful for identifying processed medicinal materials acquired in markets. Although the universality of our marker has not been sufficiently tested, it can solve our main problem. We believe that this mini-barcode method will guide related quality control research on other herbal medicines and that it will be continually applied in relevant research fields.

## Materials and methods

### Plant material preparation and sequencing

Fresh *G. sinensis* and *G. japonica* plants were picked from the garden of Tianjin University of Traditional Chinese Medicine, Tianjin, China. The voucher samples were dried and preserved in the Tianjin State Key Laboratory of Modern Chinese Medicine. A Genomic DNA extraction Kit (Sangon Biotech Co., Ltd., Shanghai, China) was used to extract the total genomic DNA. DNA purity and quantity were evaluated using a NanoPhotometer spectrophotometer (IMPLEN, CA, USA) and a Qubit 2.0 Fluorometer (Life Technologies, CA, USA), respectively. The sequencing library was generated by a Truseq Nano DNA HT Sample Preparation Kit (Illumina USA) following the manufacturer’s recommendations. The library was sequenced on Illumina HiSeq X Ten platform, and 150 bp paired-end reads were generated.

### Complete chloroplast genome construction and annotation

The total clean reads (*G. sinensis* and *G. japonica*) were filtered and assembled into contigs using GetOrganelle pipeline^63^. After that, the clean reads were re-mapped to the complete draft chloroplast genome to confirm each base, respectively. We used different tools such as DOGMA^64^, CPGAVAS2^65^, and GeSeq^66^ to annotate genes of the chloroplast genome. tRNAscan-SE^67^ was employed to verify the tRNA genes. All genes were inspected carefully against the published complete chloroplast genomes of Caesalpiniaceae (KU569489, MF741770, NC_026134, NC_028732, NC_028733, NC_034986, NC_034987, NC_034988, NC_034989, NC_034990, NC_034991, NC_034992, NC_035346, NC_035347, and NC_035348). All the start and stop codons were adjusted manually. Subsequently, the physical maps of the two complete chloroplast genome sequences were visualized with OrganellarGenomeDRAW^68^. The annotated genome sequences of *G. sinensis* and *G. japonica* were submitted to the GenBank (accession numbers: MK817503, MK817502).

### Repeated sequences and microsatellites

MISA^69^ was employed to predict single sequence repeats (SSR) or microsatellites in the complete chloroplast genome of the two *Gleditsia* species. The minimum number of repeats was set to 10, 6, 5, 5, 5, and 5 for mononucleotide, dinucleotide, trinucleotide, tetranucleotide, pentanucleotide, and hexanucleotide, respectively. We also identified forward (F), reverse (R), complementary (C), and palindromic (P) repeats using the online REPuter software^70^, with a minimum repeat size of 30 bp and a Hamming distance of 3. Tandem repeats were detected by Tandem Repeats Finder web tool^71^, using default parameters.

### Analysis and comparison of genome structures

Codon usage was determined by MEGA-X^72^. 35 protein-coding genes of *G. sinensis* and *G. japonica* chloroplast genomes were used to predict the potential RNA editing sites by the online program Predictive RNA Editor for Plants suite^73^, using default parameters. The program mVISTA^74^ in Shuffle-LAGAN mode was used to perform the structural comparison of two chloroplast genomes. At the same time, structural variations between the two *Gleditsia* chloroplast genomes were further compared by the Mauve software^75^.

### Phylogenetic analysis

Phylogenetic analysis was based on 75 shared protein-coding genes of the chloroplast genomes of 155 members of the Leguminosae family, including *G. sinensis* and *G. japonica. Rumex acetosa* (Polygonaceae) was used as an outgroup (Supplementary Table S8). Alignments were performed by MAFFT v7 with default parameters^76^. A maximum likelihood (ML) approach was used to infer phylogenetic relationships. Maximum likelihood analysis was performed using IQ-TREE v1.6.1^77^, with 1,000 bootstrap replicates. The best-fit model was determined by ModelFinder^78^.

### Analysis of sequence divergences

To analyze nucleotide diversity, we performed a sliding window analysis to assess nucleotide variability (Pi) by the DnaSP software version 6.11.01^79^. The window length was set to 600 bp, and the step size was 200 bp.

### Validation of the quantitative capacity of mini-barcode primers by metabarcoding

To verify the quantitative ability of these two primer pairs in our subjects, three mock communities were prepared, containing *G. sinensis* and *G. japonica* (Supplementary Table S9). Genomic DNA was extracted from each mock community, respectively. The target regions were amplified using two pairs of fusion primers with matching tags (e.g., F1-R1, F2-R2.) (Supplementary Table S10) to ensure that tag jumps would not result in the false assignment of sequences to samples^80^. PCR amplification was conducted in a 25 μl reaction composed of 12.5 μl of TaKaRa 2 × Gflex PCR Buffer (containing 1 mM of Mg^2+^ and 200 μM of each dNTP), 0.2 μM of each primer, 0.5 μl Tks Gflex DNA Polymerase (1.25 units/μl), approximately 10 μl ddH_2_O, and 30–50 ng DNA. The PCR protocol was as follows: preheating at 94 °C for 1 min, 30 cycles at 98 °C for 10 s, annealing at 55 °C for 15 s, and elongation at 68 °C for 30 s, followed by a final extension at 68 °C for 5 min. Negative controls were included in each run. Amplicons (including negative controls) were resolved on 1.5% agarose gels and sequenced (2 × 150 bp paired‐ends) on the Illumina Hiseq X Ten platform.

The fastq-multx^81,82^ was used to split data according to the tag sequences. Primer sequences were trimmed by BBDuk (https://sourceforge.net/projects/bbmap/). To construct ASV, denoise, and quality control (including removal of chimeras) were performed with the DADA2^83^. Meanwhile, reads were truncated to exclude low-quality data (N120 bp for forward reads and N120 bp for reverse reads, truncQ = 2, maxEE = 2). In addition, taxonomy was assigned to ASV with the chloroplast genome in our work (99% similarity at least). The relationship between biomass and individual reads was visualized for each species.

### Evaluation of the efficiency of the mini-barcode of primers ZJ818F-1038R in identifying processed medicinal materials

Two samples from different parts of *G. sinensis*, named Da Zao Jiao (fruit), Zao Jiao Ci (thorn), and one type of Chinese patent medicine (Wang Bi capsules), were purchased from the market to test the amplification ability of primers ZJ818F-1038R. PCR method was similar to the part of “Validation the quantitative capacity of mini-barcode primers by metabarcoding”. PCR products were sequenced by the Sanger method.

## Supplementary information

Supplementary Information.
