# Supplementary material for: The complete chloroplast genome of Gleditsia sinensis and Gleditsia japonica: genome organization, comparative analysis, and development of taxon specific DNA mini-barcodes
Source: Sci Rep. 2020 Oct 1;10:16309. doi: 10.1038/s41598-020-73392-7 (PMC7529812; doi:10.1038/s41598-020-73392-7)

Supporting Information

Title: The Complete Chloroplast Genome of *Gleditsia sinensis* and *Gleditsia japonica*: Genome organization, Comparative Analysis, and Development of Taxon Specific DNA Mini-barcodes

Wei Tan[1+], Han Gao[1+], Weiling Jiang[1], Huanyu Zhang[1], Xiaolei Yu [1], Erwei Liu[1,*], and Xiaoxuan Tian [1,*]

[1] Tianjin State Key Laboratory of Modern Chinese Medicine, Tianjin University of Traditional Chinese Medicine, Poyang lake road 10, 301617, Tianjin, China

[*]Corresponding: liuwei628@hotmail.com (E.L.);
tian_xiaoxuan@tjutcm.edu.cn (X.T.)

[+]These authors contributed equally to this work

Supplementary Table S1. The condon-anticodon recognition pattern and codon usage for the *G. sinensis* chloroplast genome.

Amino acid	Codon	No.	RSCU	tRNA	Amino acid	Codon	No.	RSCU	tRNA
Phe	UUU(F)	988	1.31		Tyr	UAU(Y)	783	1.6	
Phe	UUC(F)	517	0.69	trnF-GAA	Tyr	UAC(Y)	195	0.4	trnY-GUA
Leu	UUA(L)	846	1.83	trnL-UAA	Stop	UAA(*)	50	1.76	
Leu	UUG(L)	589	1.28	trnL-CAA	Stop	UAG(*)	18	0.64	
Leu	CUU(L)	582	1.26		His	CAU(H)	503	1.58	
Leu	CUC(L)	184	0.4		His	CAC(H)	132	0.42	trnH-GUG
Leu	CUA(L)	386	0.84	trnL-UAG	Gln	CAA(Q)	711	1.56	trnQ-UUG
Leu	CUG(L)	182	0.39		Gln	CAG(Q)	202	0.44	
Ile	AUU(I)	1152	1.5		Asn	AAU(N)	992	1.54	
Ile	AUC(I)	430	0.56	trnI-GAU	Asn	AAC(N)	296	0.46	trnN-GUU
Ile	AUA(I)	726	0.94	trnI-CAU	Lys	AAA(K)	1088	1.51	trnK-UUU
Met	AUG(M)	621	1	trn(f)M-CAU	Lys	AAG(K)	351	0.49	
Val	GUU(V)	511	1.45		Asp	GAU(D)	852	1.63	
Val	GUC(V)	162	0.46	trnV-GAC	Asp	GAC(D)	191	0.37	trnD-GUC
Val	GUA(V)	547	1.55	trnV-UAC	Glu	GAA(E)	1008	1.48	trnE-UUC
Val	GUG(V)	193	0.55		Glu	GAG(E)	356	0.52	
Ser	UCU(S)	577	1.71		Cys	UGU(C)	225	1.44	
Ser	UCC(S)	330	0.98	trnS-GGA	Cys	UGC(C)	88	0.56	trnC-GCA
Ser	UCA(S)	429	1.27	trnS-UGA	Stop	UGA(*)	17	0.6	
Ser	UCG(S)	179	0.53		Trp	UGG(W)	454	1	trnW-CCA
Pro	CCU(P)	419	1.58		Arg	CGU(R)	342	1.31	trnR-ACG
Pro	CCC(P)	197	0.74		Arg	CGC(R)	99	0.38	
Pro	CCA(P)	307	1.16	trnP-UGG	Arg	CGA(R)	353	1.35	
Pro	CCG(P)	139	0.52		Arg	CGG(R)	112	0.43	
Thr	ACU(T)	518	1.58		Ser	AGU(S)	400	1.18	
Thr	ACC(T)	244	0.74	trnT-GGU	Ser	AGC(S)	114	0.34	trnS-GCU
Thr	ACA(T)	415	1.26	trnT-UGU	Arg	AGA(R)	486	1.86	trnR-UCU
Thr	ACG(T)	136	0.41		Arg	AGG(R)	175	0.67	
Ala	GCU(A)	632	1.84		Gly	GGU(G)	603	1.37	
Ala	GCC(A)	209	0.61		Gly	GGC(G)	163	0.37	trnG-GCC
Ala	GCA(A)	381	1.11	trnA-UGC	Gly	GGA(G)	719	1.63	trnG-UCC
Ala	GCG(A)	152	0.44		Gly	GGG(G)	281	0.64	

Supplementary Table S2. The condon-anticodon recognition pattern and codon usage for the *Gleditsia japonica* chloroplast genome.

Amino acid	Codon	No.	RSCU	tRNA	Amino acid	Codon	No.	RSCU	tRNA
Phe	UUU(F)	991	1.31		Tyr	UAU(Y)	786	1.61	
Phe	UUC(F)	517	0.69	trnF-GAA	Tyr	UAC(Y)	193	0.39	trnY-GUA
Leu	UUA(L)	851	1.84	trnL-UAA	Stop	UAA(*)	51	1.8	

Leu	UUG(L)	588	1.27	trnL-CAA	Stop	UAG(*)	17	0.6	
Leu	CUU(L)	575	1.24		His	CAU(H)	496	1.58	
Leu	CUC(L)	186	0.4		His	CAC(H)	131	0.42	trnH-GUG
Leu	CUA(L)	389	0.84	trnL-UAG	Gln	CAA(Q)	717	1.56	trnQ-UUG
Leu	CUG(L)	183	0.4		Gln	CAG(Q)	203	0.44	
Ile	AUU(I)	1152	1.5		Asn	AAU(N)	1007	1.56	
Ile	AUC(I)	429	0.56	trnI-GAU	Asn	AAC(N)	286	0.44	trnN-GUU
Ile	AUA(I)	724	0.94	trnI-CAU	Lys	AAA(K)	1082	1.51	trnK-UUU
Met	AUG(M)	622	1	trn(f)M-CAU	Lys	AAG(K)	350	0.49	
Val	GUU(V)	514	1.46		Asp	GAU(D)	853	1.63	
Val	GUC(V)	163	0.46	trnV-GAC	Asp	GAC(D)	193	0.37	trnD-GUC
Val	GUA(V)	542	1.54	trnV-UAC	Glu	GAA(E)	1005	1.48	trnE-UUC
Val	GUG(V)	192	0.54		Glu	GAG(E)	357	0.52	
Ser	UCU(S)	578	1.71		Cys	UGU(C)	226	1.44	
Ser	UCC(S)	329	0.98	trnS-GGA	Cys	UGC(C)	87	0.56	trnC-GCA
Ser	UCA(S)	431	1.28	trnS-UGA	Stop	UGA(*)	17	0.6	
Ser	UCG(S)	173	0.51		Trp	UGG(W)	453	1	trnW-CCA
Pro	CCU(P)	420	1.58		Arg	CGU(R)	343	1.31	trnR-ACG
Pro	CCC(P)	195	0.74		Arg	CGC(R)	98	0.37	
Pro	CCA(P)	303	1.14	trnP-UGG	Arg	CGA(R)	355	1.35	
Pro	CCG(P)	143	0.54		Arg	CGG(R)	112	0.43	
Thr	ACU(T)	518	1.57		Ser	AGU(S)	396	1.17	
Thr	ACC(T)	245	0.74	trnT-GGU	Ser	AGC(S)	116	0.34	trnS-GCU
Thr	ACA(T)	416	1.26	trnT-UGU	Arg	AGA(R)	493	1.88	trnR-UCU
Thr	ACG(T)	137	0.42		Arg	AGG(R)	172	0.66	
Ala	GCU(A)	636	1.85		Gly	GGU(G)	605	1.37	
Ala	GCC(A)	206	0.6		Gly	GGC(G)	163	0.37	trnG-GCC
Ala	GCA(A)	382	1.11	trnA-UGC	Gly	GGA(G)	723	1.63	trnG-UCC
Ala	GCG(A)	154	0.45		Gly	GGG(G)	279	0.63	

Supplementary Table S3. RNA editing predicted in *Gleditsia sinensis* chloroplast genomes by the PREP program.

**Gene Name**	**Length**	**Strand**	**Region**	**Nt pos**	**AA pos**	**Align Col**	**Effect**	**Score**
accD	1491	+	LSC	341	114	121	CCA (P) => CTA (L)	1
accD	1491	+	LSC	818	273	288	TCG (S) => TTG (L)	0.8
atpA	1533	—	LSC	791	264	264	CCC (P) => CTC (L)	1
atpA	1533	—	LSC	914	305	305	TCA (S) => TTA (L)	1
clpP	588	—	LSC	556	186	187	CAT (H) => TAT (Y)	1
matK	1500	—	LSC	532	178	191	CTC (L) => TTC (F)	0.86
matK	1500	—	LSC	1184	395	408	TCA (S) => TTA (L)	0.86
ndhA	1092	—	SSC	341	114	114	TCA (S) => TTA (L)	1
ndhB	1533	+	IR	149	50	50	TCA (S) => TTA (L)	1
ndhB	1533	+	IR	467	156	156	CCA (P) => CTA (L)	1
ndhB	1533	+	IR	542	181	181	ACG (T) => ATG (M)	1

ndhB	1533	+	IR	586	196	196	CAT (H) => TAT (Y)	1
ndhB	1533	+	IR	611	204	204	TCA (S) => TTA (L)	0.8
ndhB	1533	+	IR	737	246	246	CCA (P) => CTA (L)	1
ndhB	1533	+	IR	746	249	249	TCT (S) => TTT (F)	1
ndhB	1533	+	IR	830	277	277	TCA (S) => TTA (L)	1
ndhB	1533	+	IR	836	279	279	TCA (S) => TTA (L)	1
ndhB	1533	+	IR	1112	371	371	TCA (S) => TTA (L)	1
ndhB	1533	+	IR	1255	419	419	CAT (H) => TAT (Y)	1
ndhB	1533	+	IR	1481	494	494	CCA (P) => CTA (L)	1
ndhD	1497	—	SSC	2	1	1	ACG (T) => ATG (M)	1
ndhD	1497	—	SSC	47	16	16	TCC (S) => TTC (F)	0.8
ndhD	1497	—	SSC	383	128	128	TCA (S) => TTA (L)	1
ndhD	1497	—	SSC	599	200	200	TCA (S) => TTA (L)	1
ndhD	1497	—	SSC	674	225	225	TCG (S) => TTG (L)	1
ndhD	1497	—	SSC	878	293	293	TCA (S) => TTA (L)	1
ndhD	1497	—	SSC	1298	433	433	TCA (S) => TTA (L)	0.8
ndhD	1497	—	SSC	1405	469	469	CTT (L) => TTT (F)	0.8
ndhF	2241	—	SSC	13	5	5	CAT (H) => TAT (Y)	1
ndhF	2241	—	SSC	241	81	81	CTT (L) => TTT (F)	1
ndhF	2241	—	SSC	290	97	97	TCA (S) => TTA (L)	1
ndhF	2241	—	SSC	586	196	196	CTT (L) => TTT (F)	0.8
ndhF	2241	—	SSC	2201	734	740	GCA (A) => GTA (V)	1
ndhG	531	—	SSC	166	56	56	CAT (H) => TAT (Y)	0.8
ndhG	531	—	SSC	314	105	105	ACA (T) => ATA (I)	0.8
ndhG	531	—	SSC	494	165	165	GCT (A) => GTT (V)	0.8
petB	648	+	LSC	611	204	204	CCA (P) => CTA (L)	1
psbE	252	—	LSC	214	72	72	CCT (P) => TCT (S)	1
psbF	252	—	LSC	77	26	26	TCT (S) => TTT (F)	1
psbL	117	—	LSC	2	1	1	ACG (T) => ATG (M)	1
rpoB	3213	—	LSC	338	113	113	TCT (S) => TTT (F)	1
rpoB	3213	—	LSC	551	184	185	TCA (S) => TTA (L)	1
rpoB	3213	—	LSC	566	189	190	TCG (S) => TTG (L)	1
rpoB	3213	—	LSC	2000	667	684	TCT (S) => TTT (F)	1
rpoB	3213	—	LSC	2426	809	827	TCA (S) => TTA (L)	0.86
rpoC2	4164	—	LSC	1591	531	550	CCT (P) => TTT (F)	1
rpoC2	4164	—	LSC	1592	531	550	CCT (P) => TTT (F)	1
rpoC2	4164	—	LSC	3722	1241	1453	TCA (S) => TTA (L)	0.86
rps2	711	+	LSC	248	83	83	TCA (S) => TTA (L)	1
rps14	303	—	LSC	80	27	27	TCA (S) => TTA (L)	1
rps14	303	—	LSC	149	50	53	TCA (S) => TTA (L)	1
rps16	230	—	LSC	212	71	71	TCA (S) => TTA (L)	0.83

Supplementary Table S4. RNA editing predicted in *Gleditsia japonica* chloroplast genomes by the PREP program.

Gene Name	Length	Strand	Region	Nt pos	AA pos	Align Col	Effect	Score
accD	1491	+	LSC	341	114	121	CCA (P) => CTA (L)	1
accD	1491	+	LSC	818	273	288	TCG (S) => TTG (L)	0.8
accD	1491	+	LSC	1427	476	503	CCT (P) => CTT (L)	1
atpA	1533	–	LSC	791	264	264	CCC (P) => CTC (L)	1
atpA	1533	–	LSC	914	305	305	TCA (S) => TTA (L)	1
clpP	588	–	LSC	556	186	187	CAT (H) => TAT(Y)	1
matK	1500	–	LSC	532	178	191	CTC (L) => TTC (F)	0.86
matK	1500	–	LSC	1184	395	408	TCA (S) => TTA (L)	0.86
ndhA	1092	–	SSC	341	114	114	TCA (S) => TTA (L)	1
ndhB	1533	+	IR	149	50	50	TCA (S) => TTA (L)	1
ndhB	1533	+	IR	467	156	156	CCA (P) => CTA (L)	1
ndhB	1533	+	IR	542	181	181	ACG (T) => ATG (M)	1
ndhB	1533	+	IR	586	196	196	CAT (H) => TAT (Y)	1
ndhB	1533	+	IR	611	204	204	TCA (S) => TTA (L)	0.8
ndhB	1533	+	IR	737	246	246	CCA (P) => CTA (L)	1
ndhB	1533	+	IR	746	249	249	TCT (S) => TTT (F)	1
ndhB	1533	+	IR	830	277	277	TCA (S) => TTA (L)	1
ndhB	1533	+	IR	836	279	279	TCA (S) => TTA (L)	1
ndhB	1533	+	IR	1112	371	371	TCA (S) => TTA (L)	1
ndhB	1533	+	IR	1255	419	419	CAT (H) => TAT (Y)	1
ndhB	1533	+	IR	1481	494	494	CCA (P) => CTA (L)	1
ndhD	1497	–	SSC	2	1	1	ACG (T) => ATG (M)	1
ndhD	1497	–	SSC	47	16	16	TCC (S) => TTC (F)	0.8
ndhD	1497	–	SSC	383	128	128	TCA (S) => TTA (L)	1
ndhD	1497	–	SSC	599	200	200	TCA (S) => TTA (L)	1
ndhD	1497	–	SSC	674	225	225	TCG (S) => TTG (L)	1
ndhD	1497	–	SSC	878	293	293	TCA (S) => TTA (L)	1
ndhD	1497	–	SSC	1298	433	433	TCA (S) => TTA (L)	0.8
ndhD	1497	–	SSC	1405	469	469	CTT (L) => TTT (F)	0.8
ndhF	2241	–	SSC	13	5	5	CAT (H) => TAT (Y)	1
ndhF	2241	–	SSC	241	81	81	CTT (L) => TTT (F)	1
ndhF	2241	–	SSC	290	97	97	TCA (S) => TTA (L)	1
ndhF	2241	–	SSC	586	196	196	CTT (L) => TTT (F)	0.8
ndhF	2241	–	SSC	2201	734	740	GCA (A) => GTA (V)	1
ndhG	531	–	SSC	166	56	56	CAT (H) => TAT (Y)	0.8
ndhG	531	–	SSC	314	105	105	ACA (T) => ATA (I)	0.8
ndhG	531	–	SSC	494	165	165	GCT (A) => GTT (V)	0.8
petB	648	+	LSC	611	204	204	CCA (P) => CTA (L)	1
psbE	252	–	LSC	214	72	72	CCT (P) => TCT (S)	1
psbF	252	–	LSC	77	26	26	TCT (S) => TTT (F)	1

psbL	117	—	LSC	2	1	1	ACG (T) => ATG (M)	1
rpoB	3213	—	LSC	338	113	113	TCT (S) => TTT (F)	1
rpoB	3213	—	LSC	551	184	185	TCA (S) => TTA (L)	1
rpoB	3213	—	LSC	566	189	190	TCG (S) => TTG (L)	1
rpoB	3213	—	LSC	2000	667	684	TCT (S) => TTT (F)	1
rpoB	3213	—	LSC	2426	809	827	TCA (S) => TTA (L)	0.86
rpoC1	2049	—	LSC	41	14	14	TCA (S) => TTA (L)	1
rpoC2	4164	—	LSC	1591	531	550	CCT (P) => TTT (F)	1
rpoC2	4164	—	LSC	1592	531	550	CCT (P) => TTT (F)	1
rpoC2	4164	—	LSC	3722	1241	1453	TCA (S) => TTA (L)	0.86
rps2	711	+	LSC	248	83	83	TCA (S) => TTA (L)	1
rps14	303	—	LSC	80	27	27	TCA (S) => TTA (L)	1
rps14	303	—	LSC	149	50	53	TCA (S) => TTA (L)	1

Supplementary Table S5. Overview of Illumina sequencing data.

	**Raw reads**	**Clean reads**		**ASV**		Unclassified reads(%)
	**Total**	**Reads after quality control and denoising**	**Non-chimeric reads**	**Total**	**The number of assigned ASV**	
818F-1037R	780337	730189	707894	3	3	0
1118F-1287R	769474	711352	686814	5	3	0.014

Supplementary Table S6. The first column is the identified species, the remaining columns are the ASV's counts of two pairs of primers in three mock communities.

**species**	**reads count**					
	ZJ818F -1038R			ZJ1118F-1287R		
	ZJ1	ZJ2	ZJ3	ZJ1	ZJ2	ZJ3
G. sinensis	289334	353	87254	109231	43	525031
G.japonica	294154	36145	654	48199	2979	1331

Supplementary Table S7. Identification result of the mini-barcode of primer ZJ818F-1038R in three processed medicinal materials.

**Sample**	**identification**	**identity**	**Sequence**
Da Zao Jiao	*G. sinensis*	99.47 %	AATTAAACAAGATAAAAAAAACTACAATGAAGAAGATCTTTCTACTTC TATTTTTTCAGAAAAAAGGGAGGATTTGTACAAAATCGATGAAAGAG AAGAGAAAGATATCTTCCGATTGGAAAAACCTCTTCTAAAAATCCTTT TCGATTATAAACGATTCCATCGTCCATTGCGATATATAAAAAATAG
Zao Jiao Ci	*G. sinensis*	100%	AATTAAACAAGATAAAAAAAACTACAATGAAGAAGATCTTTCTCCTTC TATTTTTTCAGAAAAAAGGGAGGATTTGTACAAAATCGATGAAAGAG AAGAGAAAGATATCTTCCGATTGGAAAAACCTCTTCTAAAAATCCTTT TCGATTATAAACGATTCCATCGTCCATTGCGATATATAAAAAATAG

Wang Bi capsules	*G. sinensis*	100%	AATTAAACAAGATAAAAAAAACTACAATGAAGAAGATCTTTCTCCTTC TATTTTTTCAGAAAAAAGGGAGGATTTGTACAAAATCGATGAAAGAG AAGAGAAAGATATCTTCCGATTGGAAAAACCTCTTCTAAAAATCCTTT TCGATTATAAACGATTCCATCGTCCATTGCGATATATAAAAAATAG

Supplementary Table S8. The list of accession numbers of the chloroplast genome sequences was used phylogenetic analysis.

**number**	**Taxon**	**accession numbers**
1	*Acacia dealbata*	NC_034985
2	*Acacia ligulata*	NC_026134
3	*Adenanthera microsperma*	NC_034986
4	*Adenolobus garipensis*	NC_036761
5	*Albizia odoratissima*	NC_034987
6	*Ammopiptanthus mongolicus*	NC_034742
7	*Ammopiptanthus nanus*	NC_034743
8	*Apios americana*	NC_025909
9	*Arachis hypogaea*	NC_037358
10	*Archidendron lucyi*	NC_034988
11	*Astragalus membranaceus var membranaceus*	KX255662
12	*Astragalus mongholicus*	KU666554
13	*Astragalus mongholicus var nakaianus*	NC_028171
14	*Balsamocarpon brevifolium*	NC_041581
15	*Barklya syringifolia*	NC_037761
16	*Bauhinia acuminata*	NC_037762
17	*Bauhinia binata*	NC_037764
18	*Cajanus cajan*	NC_031429
19	*Cajanus scarabaeoides*	KU729878
20	*Campylotropis macrocarpa*	NC_044100
21	*Caragana korshinskii*	NC_035229
22	*Caragana kozlowii*	NC_035228
23	*Caragana microphylla*	NC_032691
24	*Caragana rosea var rosea*	NC_039932
25	*Ceratonia siliqua*	NC_026678
26	*Cercis canadensis*	KF856619
27	*Cercis chuniana*	MF741770
28	*Cercis glabra*	NC_036762
29	*Cicer arietinum*	NC_011163
30	*Crudia harmsiana*	NC_036743
31	*Cyamopsis tetragonoloba*	NC_037714
32	*Dalbergia cultrata*	NC_044117
33	*Dalbergia hainanensis*	NC_036961
34	*Dalbergia odorifera*	MF668133
35	*Daniellia pilosa*	NC_036744
36	*Desmodium heterocarpon*	NC_044113

37	*Dichrostachys cinerea*	NC_035346
38	*Erythrophleum fordii*	NC_041164
39	*Faidherbia albida*	NC_035347
40	*Glycine canescens*	NC_021647
41	*Glycine cyrtoloba*	NC_021645
42	*Glycine dolichocarpa*	NC_021648
43	*Glycine falcata*	NC_021649
44	*Glycine gracilis*	NC_030329
45	*Glycine max*	NC_007942
46	*Glycine soja*	KY241814
47	*Glycine stenophita*	NC_021646
48	*Glycine syndetika*	NC_021650
49	*Glycine tomentella*	NC_021636
50	*Glycyrrhiza glabra*	NC_024038
51	*Glycyrrhiza glabra x Glycyrrrhiza uralensis*	KU862307
52	*Glycyrrhiza inflata*	NC_042146
53	*Glycyrrhiza lepidota*	NC_034229
54	*Glycyrrhiza uralensis*	KU862308
55	*Griffonia simplicifolia*	NC_037763
56	*Guibourtia leonensis*	NC_036742
57	*Haematoxylum brasiletto*	NC_026679
58	*Hylodesmum podocarpum subsp podocarpum*	MG867568
59	*Indigofera tinctoria*	NC_026680
60	*Inga leiocalycina*	NC_028732
61	*Kummerowia striata*	NC_044114
62	*Lathyrus clymenum*	NC_027148
63	*Lathyrus davidii*	NC_027073
64	*Lathyrus graminifolius*	NC_027074
65	*Lathyrus inconspicuus*	NC_027149
66	*Lathyrus japonicus*	NC_027075
67	*Lathyrus littoralis*	NC_027076
68	*Lathyrus ochroleucus*	NC_027077
69	*Lathyrus odoratus*	NC_027150
70	*Lathyrus palustris*	KJ806199
71	*Lathyrus pubescens*	NC_027079
72	*Lathyrus sativus*	NC_014063
73	*Lathyrus tingitanus*	NC_027151
74	*Lathyrus venosus*	NC_027080
75	*Lens culinaris*	NC_027152
76	*Lespedeza davurica*	NC_042748
77	*Lespedeza floribunda*	NC_042693
78	*Lespedeza maritima*	NC_044115
79	*Lessertia frutescens*	NC_036151
80	*Leucaena trichandra*	NC_028733

81	*Libidibia coriaria*	NC_026677
82	*Lotus japonicus*	NC_002694
83	*Lupinus albus*	NC_026681
84	*Lupinus atlanticus*	KU726827
85	*Lupinus luteus*	KC695666
86	*Lupinus micranthus*	KU726828
87	*Lupinus princei*	KU726829
88	*Lupinus westianus*	NC_036487
89	*Lysiphyllum hookeri*	NC_037768
90	*Maackia floribunda*	NC_034774
91	*Medicago falcata*	NC_032066
92	*Medicago hybrida*	NC_027153
93	*Medicago papillosa*	NC_027154
94	*Medicago sativa*	KU321683
95	*Medicago truncatula*	NC_003119
96	*Medicago truncatula f tricycla*	KF241982
97	*Melilotus albus*	NC_041419
98	*Mezoneuron cucullatum*	KU569489
99	*Millettia pinnata*	NC_016708
100	*Mimosa pudica*	NC_042921
101	*Mucuna macrocarpa*	NC_044116
102	*Ohwia caudata*	NC_044105
103	*Ormosia hosiei*	NC_039418
104	*Pachyrhizus erosus*	NC_026682
105	*Pararchidendron pruinosum*	NC_035348
106	*Parkia javanica*	NC_034989
107	*Phaseolus vulgaris*	NC_009259
108	*Piliostigma thonningii*	NC_037765
109	*Piptadenia communis*	NC_034990
110	*Pisum abyssinicum*	NC_037830
111	*Pisum fulvum*	NC_036828
112	*Pisum sativum*	NC_014057
113	*Pithecellobium flexicaule*	NC_034991
114	*Prosopis glandulosa*	NC_026683
115	*Pterocarpus indicus*	MH033831
116	*Pterocarpus santalinus*	MH033830
117	*Pterocarpus tinctorius*	MH033829
118	*Robinia pseudoacacia*	NC_026684
119	*Rumex acetosa* (outgroup)	NC_042390
120	*Salweenia bouffordiana*	MF449303
121	*Samanea saman*	NC_034992
122	*Schnella trichosepala*	NC_037766
123	*Senegalia laeta*	NC_036736
124	*Senna occidentalis*	NC_038222

125	*Senna tora*	NC_030193
126	*Sophora alopecuroides*	NC_036102
127	*Sophora flavescens*	MH748034
128	*Sophora tonkinensis*	NC_042688
129	*Stryphnodendron adstringens*	MN196294
130	*Stylosanthes scabra*	NC_039160
131	*Stylosanthes viscosa*	NC_039161
132	*Styphnolobium japonicum f violaceum*	KY872756
133	*Tamarindus indica*	NC_026685
134	*Tibetia liangshanensis*	NC_036109
135	*Trifolium boissieri*	NC_025743
136	*Trifolium glanduliferum*	NC_025744
137	*Trifolium meduseum*	NC_024166
138	*Trifolium strictum*	NC_025745
139	*Trifolium subterraneum*	NC_011828
140	*Tylosema fassoglense*	NC_037767
141	*Vachellia flava*	NC_036734
142	*Vachellia nilotica subsp*	KY100264
143	*Vachellia seyal*	NC_036735
144	*Vachellia tortilis subsp raddiana*	KY100266
145	*Vicia sativa*	NC_027155
146	*Vicia sepium*	NC_039595
147	*Vigna angularis*	NC_021091
148	*Vigna radiata*	NC_013843
149	*Vigna unguiculata*	NC_018051
150	*Wisteria floribunda*	KM103376
151	*Wisteria sinensis*	NC_029406
152	*Gleditsia japonica*	MK817502
153	*Gleditsia sinensis*	MK817503
154	*Gleditsia microphylla*	MN709853
155	*Duparquetia orchidacea*	MN709829
156	*Zenia insignis*	NC_045299

Supplementary Table S9. Estimated biomass for each species included in the mock communities (ZJ1-3). Values are presented in milligrams (mg).

	**Biomass (mg)**		
	**mork community**		
**Species**	**ZJ1**	**ZJ2**	**ZJ3**
Gleditsia sinensis	54.6	1.1	99.1
Gleditsia japonica	52.0	99.5	1.3

Supplementary Table S10. Tag sequences of two primers in three mork communities.

**primers**	**Tag sequences**		
	**ZJ1**	**ZJ2**	**ZJ3**

ZJ818F-1038R	For (5'-3') TAGCGTCT	For (5'-3') TCTGCATC	For (5'-3') TCATGTGC
	Rev (5'-3') TAGCGTCT	Rev (5'-3') TCTGCATC	Rev (5'-3') TCATGTGC
ZJ1118F-1287R	For (5'-3') TCACTACG	For (5'-3') TCGTAGCA	For (5'-3') TGAGACGT
	Rev (5'-3') TCACTACG	Rev (5'-3') TCGTAGCA	Rev (5'-3') TGAGACGT

Supplementary Fig. S1. The result of the chloroplast genome structure comparison using MAUVE.

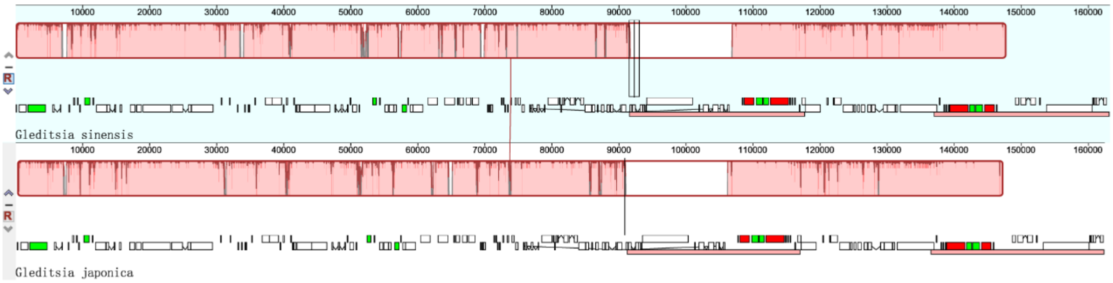

Supplementary Fig. S2. PCR amplification profile of mini-barcode of primer ZJ818F-1038R of three processed medicinal materials. PCR bands from left to right are Da Zao Jiao, Zao Jiao Ci, Wang Bi capsules and negative control.

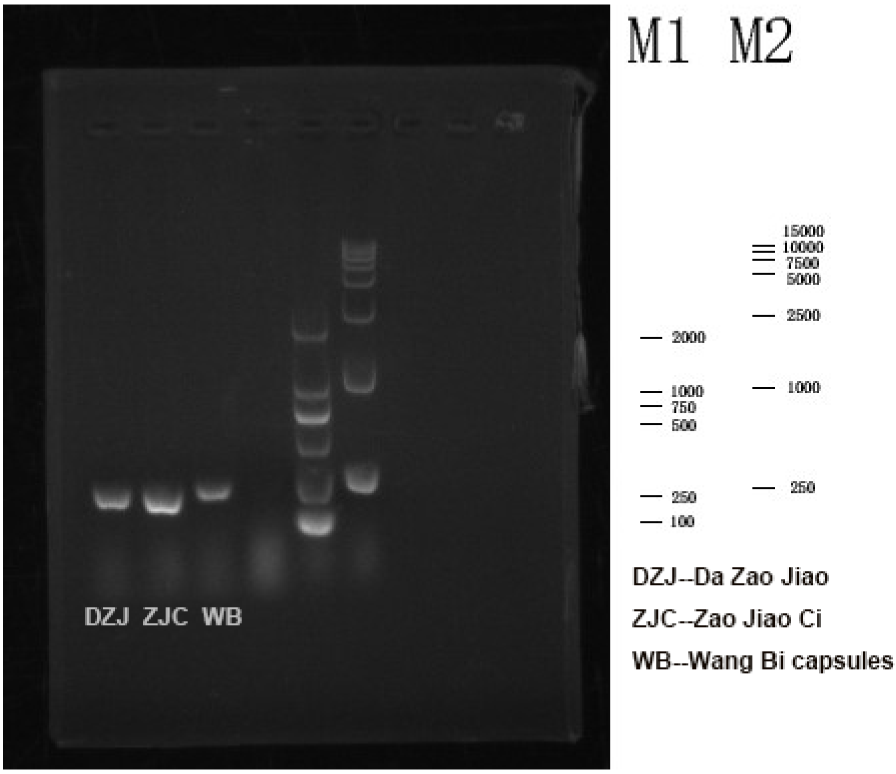

Supplement: Supplementary file 1 — Supplementary Information. [file 41598_2020_73392_MOESM1_ESM.pdf]
